# A generalized reward processing deficit pathway to negative symptoms across diagnostic boundaries

**DOI:** 10.1017/S003329172400326X

**Published:** 2025-02-04

**Authors:** Michael J. Spilka, Zachary B. Millman, James A. Waltz, Elaine F. Walker, Jason A. Levin, Albert R. Powers, Philip R. Corlett, Jason Schiffman, James M. Gold, Steven M. Silverstein, Lauren M. Ellman, Vijay A. Mittal, Scott W. Woods, Richard Zinbarg, Gregory P. Strauss

**Affiliations:** 1 Department of Psychology, University of Georgia, Athens, GA, USA; 2 Psychotic Disorders Division, McLean Hospital, Belmont, MA, USA; 3 Department of Psychiatry, Harvard Medical School, Boston, MA, USA; 4 Maryland Psychiatric Research Center, Department of Psychiatry, University of Maryland School of Medicine, Baltimore, MD, USA; 5 Department of Psychology, Emory University, Atlanta, GA, USA; 6 Department of Psychiatry, Yale University, New Haven, CT, USA; 7 Department of Psychological Science, University of California, Irvine, CA, USA; 8 Departments of Psychiatry, Neuroscience and Ophthalmology, University of Rochester Medical Center, Rochester, NY, USA; 9 Department of Psychology & Neuroscience, Temple University, Philadelphia, PA, USA; 10 Department of Psychology, Northwestern University, Evanston, IL, USA

**Keywords:** clinical high-risk, equifinality, negative symptoms, psychosis, reward, transdiagnostic

## Abstract

**Background:**

Negative symptoms are a key feature of several psychiatric disorders. Difficulty identifying common neurobiological mechanisms that cut across diagnostic boundaries might result from equifinality (i.e., multiple mechanistic pathways to the same clinical profile), both within and across disorders. This study used a data-driven approach to identify unique subgroups of participants with distinct reward processing profiles to determine which profiles predicted negative symptoms.

**Methods:**

Participants were a transdiagnostic sample of youth from a multisite study of psychosis risk, including 110 individuals at clinical high-risk for psychosis (CHR; meeting psychosis-risk syndrome criteria), 88 help-seeking participants who failed to meet CHR criteria and/or who presented with other psychiatric diagnoses, and a reference group of 66 healthy controls. Participants completed clinical interviews and behavioral tasks assessing four reward processing constructs indexed by the RDoC Positive Valence Systems: hedonic reactivity, reinforcement learning, value representation, and effort–cost computation.

**Results:**

*k*-means cluster analysis of clinical participants identified three subgroups with distinct reward processing profiles, primarily characterized by: a value representation deficit (54%), a generalized reward processing deficit (17%), and a hedonic reactivity deficit (29%). Clusters did not differ in rates of clinical group membership or psychiatric diagnoses. Elevated negative symptoms were only present in the generalized deficit cluster, which also displayed greater functional impairment and higher psychosis conversion probability scores.

**Conclusions:**

Contrary to the equifinality hypothesis, results suggested one global reward processing deficit pathway to negative symptoms independent of diagnostic classification. Assessment of reward processing profiles may have utility for individualized clinical prediction and treatment.

## Introduction

Negative symptoms, though historically conceptualized within psychotic disorders, are now recognized to have a transdiagnostic presentation within a wide range of psychiatric illnesses, including mood, anxiety, and trauma-related syndromes (Strauss & Cohen, 2017). Transdiagnostic studies of the neurobehavioral mechanisms thought to give rise to these phenomena have the potential to reveal treatment targets across diagnostic categories. Such approaches may be especially valuable in the early and high-risk stages of psychopathology, when neurodevelopment is still underway, pathogenic mechanisms are presumably forming, and secondary prevention of adverse clinical outcomes is possible (McGorry et al., 2014). Growing evidence indicates that youth at clinical high risk (CHR) for psychosis present with abnormalities in laboratory measures of reward processing, and these abnormalities are often associated with negative symptom severity (Bartolomeo, Chapman, Raugh, & Strauss, 2021; Strauss, Bartolomeo, & Luther, 2023). However, the extent to which reward-related behavioral alterations in youth at CHR diverge or overlap with those seen among help-seeking peers not at CHR for psychosis remains unknown, representing a key limitation in our understanding of negative symptom etiology (Millman, Gold, Mittal, & Schiffman, 2019; Strauss & Cohen, 2017).

The National Institute of Mental Health’s Research Domain Criteria (RDoC) provides a useful framework for studying negative symptoms across diagnostic categories, where the Positive Valence Systems domain includes dissociable aspects of reward processing that contribute to motivated behavior (Cuthbert, 2014). For example, individuals with schizophrenia, depression, and bipolar disorder often show qualitatively similar alterations in reward valuation, including reduced willingness to exert effort for rewards (both cognitive and physical; Culbreth, Moran, & Barch, 2018; Hershenberg et al., 2016; Zou et al., 2020), and greater discounting of the value of delayed versus immediate rewards (Amlung et al., 2019). However, accumulating evidence within this framework has also highlighted heterogeneity in reward processing dysfunction across diagnostic boundaries (e.g., hedonic reactivity is altered in major depressive disorder and spared in schizophrenia; explicit reinforcement learning is relatively impaired in schizophrenia and spared in major depression; implicit reinforcement learning is relatively impaired in depression and spared in schizophrenia; Barch et al., 2017; Bylsma, Morris, & Rottenberg, 2008; Cohen & Minor, 2010; Gold et al., 2012; Whitmer, Frank, & Gotlib, 2012). Anxiety, trauma, substance use, and obsessive-compulsive disorders are also associated with overlapping and divergent patterns of reward-processing behavior (Amlung et al., 2019; Bishop & Gagne, 2018; Kanen, Ersche, Fineberg, Robbins, & Cardinal, 2019; Weaver et al., 2020). The noted heterogeneity in reward processing dysfunction across disorders suggests that there may be equifinality (i.e., multiple mechanistic pathways to a given clinical outcome) in negative symptoms despite their transdiagnostic presentation (Nusslock & Alloy, 2017; Strauss & Cohen, 2017; Whitton, Treadway, & Pizzagalli, 2015).

Among youth at CHR, evidence to date suggests that the profile of reward-related abnormalities may overlap with both psychotic (e.g., altered reward learning, reduced effort expenditure) and mood (e.g., diminished hedonic reactivity, reduced effort expenditure) disorders (Gruber, Strauss, Dombrecht, & Mittal, 2018; Millman et al., 2020; Strauss et al., 2018; Strauss et al., 2023) while also possessing distinct areas of intact performance (e.g., intact delay discounting; Bartolomeo et al., 2021). The emerging picture that youth at CHR present with reward-processing dysfunction that shows both overlap and divergence with other psychiatric syndromes suggests the possibility that equifinality might also occur *within* clinical syndromes and diagnostic categories. Therefore, clinically based comparisons of multiple diagnostic groups may be limited in their ability to capture the full range of mechanistic heterogeneity underlying negative symptoms.

Data-driven stratification approaches based on multiple measures of reward-related behavior may hold promise in parsing mechanistic heterogeneity by identifying subgroups with more homogenous reward-processing profiles, with the potential to inform etiological models and facilitate individualized treatments (Marquand, Wolfers, Mennes, Buitelaar, & Beckmann, 2016; Mittal, Walker, & Strauss, 2021). Findings from such approaches may be particularly generalizable when drawn from transdiagnostic samples of help-seeking youth, including youth at CHR as well as those with common nonpsychotic disorders (Millman et al., 2019). Although recent work has demonstrated the utility of similar strategies using neuroanatomical and clinical data in CHR and clinical comparison groups (Dean, Walther, Bernard, & Mittal, 2018; Dwyer et al., 2022; Gupta, Cowan, Strauss, Walker, & Mittal, 2021; Healey et al., 2018; Koutsouleris et al., 2021; Millman et al., 2022; Ryan et al., 2018), little research has sought to identify behavioral profiles of reward processing in transdiagnostic high-risk samples. To our knowledge, this approach has only been applied to a sample that additionally included participants with more serious mental illness (e.g., schizophrenia). Luther and colleagues (Luther, Jarvis, Spilka, & Strauss, 2024) used cluster analysis to identify three distinct profiles of reward-processing task performance in a combined sample of participants with CHR, schizophrenia, schizoaffective, bipolar disorder, and psychotic-like experiences. The reward-processing profiles consisted of clusters of participants with either preserved performance across reward processing domains, isolated hedonic reactivity deficits, or a more global deficit spanning across all domains. Only the global deficit cluster demonstrated elevated negative symptoms, suggesting the presence of a single transdiagnostic and transphasic pathway to negative symptoms. However, although CHR participants were equally represented across identified clusters, the inclusion of participants with a severe mental illness diagnosis in addition to a sample of youth at CHR may have influenced the observed cluster-level negative symptom severity scores and masked more subtle variation in negative symptom patterns in the CHR group. Therefore, additional research focusing on high-risk samples is needed to clarify negative symptom pathways in the at-risk phase of illness.

The goal of this study was to use a data-driven approach to identify unique subgroups of clinically diverse youth from their performance on tasks assessing four aspects of reward processing believed to contribute to negative symptoms (Strauss & Cohen, 2017; Strauss, Waltz, & Gold, 2014) and consistent with the RDoC Positive Valence Systems constructs: hedonic reactivity (initial response to reward), reinforcement learning (probabilistic reinforcement learning), reward valuation (delay discounting), and effort–cost computation (effort valuation). Distinct participant clusters were then compared on external validators not included in the cluster analysis to determine whether clusters displayed distinct profiles of clinical and demographic features. We hypothesized that equifinality would be present in the mechanistic pathways to negative symptoms, both across and within clinical groups, as indicated by distinct reward processing profiles among clusters that show clinically significant elevations in negative symptoms but do not differ in the proportion of CHR versus other clinical cases.

## Methods and materials

### Participants

Participants were 282 individuals 15–34 years of age from the Computerized Assessment of Psychosis Risk (CAPR) study conducted across several university research sites in the USA (Mittal et al., 2021). Participants belonged to one of three groups: (1) 120 individuals at CHR for psychosis, who met progressive or persistent psychosis-risk syndrome criteria on the Structured Interview for Psychosis-Risk Syndromes (SIPS; McGlashan et al., 2001); (2) 91 help-seeking clinical comparison participants (HSC) who were referred/self-referred for psychosis-risk symptoms and/or had at least one current DSM-5 (American Psychiatric Association, 2013) nonpsychotic disorder diagnosis but did not meet criteria for a SIPS psychosis-risk syndrome; and (3) 71 healthy control (HC) participants without any current or lifetime DSM-5 psychiatric diagnoses. CHR and HSC participants were recruited through targeted online and print advertisements, email, and through contact with mental health providers in hospitals, community mental health centers, and school settings. HC participants were recruited through online and print advertisements, and email.

See the Supplementary Materials and Mittal, Ellman, et al. (2021) for detailed participant eligibility criteria, recruitment, and testing procedures.

All participants provided written consent and/or assent for a single-site protocol approved by the Northwestern University Institutional Review Board.

### Measures

A series of clinical interviews, questionnaires, cognitive tests, and computerized experimental tasks were completed online via videoconference and administered by trained research staff.

Eligibility and group membership were confirmed via the SIPS and the Structured Clinical Interview for DSM-5 (SCID; First et al., 2016). Negative symptom severity in CHR and HSC participants was assessed with the Negative Symptom Inventory – Psychosis Risk (NSI-PR; Strauss et al., 2023), a clinician-rated scale designed to evaluate negative symptom severity in CHR populations. Additional measures are listed in the Supplementary Materials.

### Experimental tasks

Participants completed an online battery of experimental tasks assessing distinct aspects of reward processing that are believed to contribute to negative symptoms (Strauss et al., 2014; Strauss & Cohen, 2017): (1) a hedonic reactivity task requiring self-report ratings of positive valence in response to pleasant images from the International Affective Picture System (IAPS; Lang, Bradley, & Cuthbert, 1997); (2) a probabilistic reinforcement learning task (PRLT; Gold et al., 2012) assessing explicit learning from gains and losses; (3) a value representation task assessing the subjective value of a hypothetical future reward as a function of its magnitude and delay in delivery (delay discounting task; Kirby, Petry, & Bickel, 1999); and (4) an effort–cost computation task in which participants had to choose between completing a lower effort task for a smaller reward or a higher effort task for a larger reward (effort expenditure for reward task; EEfRT; Treadway et al., 2012). Task paradigms assessing these reward-processing constructs were selected based on prior evidence of performance deficits in CHR and/or schizophrenia samples (Barch et al., 2017; Bartolomeo et al., 2021; Strauss et al., 2023; Strauss, Ruiz, Visser, Crespo, & Dickinson, 2018). For the reinforcement learning and effort–cost computation tasks, participants in each group were randomized to task versions using either points-based or monetary rewards, and performance differences between task versions were examined to rule out systematic differences (described below). See the Supplementary Materials for a description of each task and the computation of dependent variables.

### Data processing and sample characteristics

Data for each task were inspected to identify and exclude corrupted data or participants with inappropriate response patterns, leading to the exclusion of 10 CHR, 3 HSC, and 5 HC participants (see Supplementary Materials). Only participants with complete data across the four tasks were retained, resulting in a final sample of 110 CHR, 88 HSC, and 66 HC participants (see Table 1 for group characteristics). The HSC group on average had significantly higher years of completed education than the CHR group, but groups otherwise had similar demographic characteristics. Current DSM-5 diagnoses were present in a majority of CHR and HSC participants; however, clinical severity was greater in the CHR than HSC group across several diagnoses and clinical measures (Table 1).

**Table 1.** Participant sample

	Clinical high-risk	Help-seeking control	Healthy control	Test statistic
*N*	110	88	66	
Demographic variables				
Age	23.51 (4.26)	24.07 (4.20)	22.85 (3.91)	*F*(2,261) = 1.64, *p* = 0.196, *η*_p_^2^ = 0.01
Sex (M/F)	30/80	26/62	22/44	*χ*^2^(2, *N* = 264) = 0.73, *p* = 0.695
Ethnicity/race				*χ*^2^(10, *N* = 263) = 14.52, *p* = 0.150
Asian	17.43%	21.59%	28.78%	
Black	11.93%	13.64%	18.18%	
Latinx	13.76%	7.95%	3.03%	
Multiracial	13.76%	5.68%	6.06%	
Middle Eastern	2.75%	3.41%	1.52%	
White – non-hispanic	40.37%	47.73%	42.42%	
Education (years)	14.17 (2.16)	14.94 (2.17)	14.77 (2.08)	*F*(2,254) = 3.42, *p* = 0.034, *η*_p_^2^ = 0.03; CHR < HSC (*p* = 0.04)
Clinical variables				
NSI-PR diminished motivation and pleasure factor	1.66 (0.85)	1.30 (0.70)		*F*_W_(1,190.6) = 10.71, *p* = 0.001, *η*_p_^2^ = 0.05
NSI-PR diminished expression factor	1.02 (1.10)	0.71 (0.84)		*F*_W_(1,188.9) = 4.93, *p* = 0.028, *η*_p_^2^ = 0.02
NSI-PR anhedonia	1.31 (1.10)	1.09 (0.94)		*F*(1,190) = 2.14, *p* = 0.145, *η*_p_^2^ = 0.01
NSI-PR asociality	1.86 (1.05)	1.51 (0.88)		*F*(1,191) = 6.03, *p* = 0.015, *η*_p_^2^ = 0.03
NSI-PR avolition	1.70 (0.99)	1.19 (0.87)		*F*(1,191) = 13.75, *p* < 0.001, *η*_p_^2^ = 0.07
NSI-PR blunted affect	1.16 (1.27)	0.80 (0.93)		*F*_W_(1,188.2) = 5.26, *p* = 0.023, *η*_p_^2^ = 0.03
NSI-PR alogia	0.62 (0.91)	0.45 (0.83)		*F*(1,189) = 1.67, *p* = 0.198, *η*_p_^2^ = 0.01
Proxy primary negative symptom score (*z*-score)^a^	−0.10 (1.18)	0.13 (0.68)		*F*_W_(1,176.0) = 2.96, *p* = 0.087, *η*_p_^2^ = 0.01
SIPS positive (Total)	10.68 (3.30)	4.01 (2.67)		*F*(1,191) = 228.64, *p* < 0.001, *η*_p_^2^ = 0.55
CES-D	19.17 (9.16)	15.98 (9.10)	8.21 (5.24)	*F*(1,194) = 5.91, *p* = 0.016, *η*_p_^2^ = 0.03
PSS	30.14 (9.12)	27.68 (8.35)	20.62 (7.55)	*F*(1,194) = 3.79, *p* = 0.053, *η*_p_^2^ = 0.02
STAI (Trait)	17.44 (5.77)	15.32 (5.29)	10.89 (3.62)	*F*(1,194) = 7.09, *p* = 0.008, *η*_p_^2^ = 0.04
Any DSM–5 diagnosis (%)	80.00%	64.77%	0%	*χ*^2^(1, *N* = 198) = 5.03, *p* = 0.025
Bipolar disorders	11.82%	1.14%	0%	*χ*^2^(1, *N* = 198) = 6.94, *p* = 0.008
Depressive disorders	30.91%	30.68%	0%	*χ*^2^(1, *N* = 198) < 0.001, *p* = 1
Substance use disorders	22.73%	12.50%	0%	*χ*^2^(1, *N* = 198) = 2.78, *p* = 0.095
Anxiety disorders	66.36%	48.86%	0%	*χ*^2^(1, *N* = 198) = 5.47, *p* = 0.019
Obsessive–compulsive disorders	10.91%	6.82%	0%	*χ*^2^(1, *N* = 198) = 0.56, *p* = 0.455
Trauma disorders	32.73%	13.64%	0%	*χ*^2^(1, *N* = 198) = 8.69, *p* = 0.003
Global functioning: Social	7.33 (1.37)	7.85 (1.28)	8.60 (0.97)	*F*_W_(1,180.0) = 7.27, *p* = 0.008, *η*_p_^2^ = 0.04
Global functioning: Role	7.58 (1.53)	8.41 (0.98)	9.08 (0.71)	*F*(1,188) = 18.45, *p* < 0.001, *η*_p_^2^ = 0.09
NAPLS 1-year psychosis conversion risk probability	8.75 (5.29)			
NAPLS 2-year psychosis conversion risk probability	11.50 (6.78)			
Current antipsychotic use	5.61%	1.16%	0%	*χ*^2^(1, *N* = 193) = 1.57, *p* = 0.210
Cognitive variables				
Estimated premorbid functioning (WRAT–4 word reading)	110.10 (15.05)	112.24 (11.34)	110.93 (11.86)	*F*(2,218) = 0.54, *p* = 0.581, *η*_p_^2^ = 0.01
Verbal learning (HVLT) Total score	26.72 (4.99)	27.51 (4.80)	27.40 (4.52)	*F*(2,251) = 0.74, *p* = 0.477, *η*_p_^2^ = 0.01
Verbal learning (HVLT) Learning rate (Trial 3–Trial 1)	2.94 (1.97)	2.48 (1.39)	2.75 (1.78)	*F*(2,251) = 1.63, *p* = 0.199, *η*_p_^2^ = 0.01
Processing speed (BACS symbol coding)	58.87 (12.75)	58.88 (12.64)	60.22 (10.87)	*F*(2,229) = 0.27, *p* = 0.767, *η*_p_^2^ = 0.00
Task performance				
Hedonic reactivity (average IAPS pleasantness rating)	4.01 (0.69)	4.03 (0.60)	4.07 (0.58)	*F*(2,261) = 0.21, *p* = 0.811, *η*_p_^2^ = 0.00
Value representation (average *k*-delay discounting rate^b^)	−4.25 (1.72)	−4.48 (1.63)	−4.61 (1.57)	*F*(2,261) = 1.09, *p* = 0.338, *η*_p_^2^ = 0.01
Effort–cost computation (EEfRT % hard task choices^c^)	0.56 (0.26)	0.62 (0.28)	0.60 (0.29)	*F*(2,261) = 1.57, *p* = 0.210, *η*_p_^2^ = 0.01
Reinforcement learning (PRLT % gain condition accuracy^d^)	0.85 (0.15)	0.86 (0.15)	0.84 (0.14)	*F*(2,261) = 0.32, *p* = 0.723, *η*_p_^2^ = 0.00

*Note.* Values reflect mean (standard deviation) unless otherwise indicated. Statistical comparisons for demographic variables and task performance were performed across all groups. Statistical comparisons for clinical variables were performed for clinical high-risk and help-seeking control groups.

BACS = Brief Assessment of Cognition in Schizophrenia; CES-D = Center for Epidemiologic Studies – Depression Scale; EEfRT = Effort Expenditure for Rewards Task; HVLT = Hopkins Verbal Learning Test; IAPS = International Affective Picture System; NSI-PR = Negative Symptom Inventory – Psychosis Risk; PSS = Perceived Stress Scale; PRLT = Probabilistic Reinforcement Learning Task; SIPS = Structured Interview for Psychosis-Risk Syndromes; STAI = State-Trait Anxiety Inventory; WRAT-4 = Wide-Range Achievement Test – 4.

a Proxy primary negative symptom score is the scaled difference score of N1 + N2 + N3 - G2 - G4 SIPS items.

b Reported values are log-transformed.

c Percentage of hard task choices for trials in the upper half of the reward magnitude range and in the upper (50%–88%) reward probability range.

d Percentage of correct choices on the most frequently rewarded gain stimulus pairing across learning trials.

### Cluster analysis

In order to facilitate interpretations of task performance deficits, particularly in the context of participants completing the tasks in a remotely administered format and during the course of the COVID-19 pandemic, CHR and HSC task performance scores were standardized relative to those of the HC group prior to analysis, which served as a normative sample to evaluate performance (delay discounting signs were also reversed to facilitate interpretation). The standardized scores for CHR and HSC participants were then subjected to a cluster analysis using *k*-means clustering (Steinley, 2006), with squared Euclidean distance as the dissimilarity measure (starting configurations = 50, maximum iterations = 100). As a verification step, a similar cluster analysis was performed using the nonreferenced CHR and HSC performance scores (i.e., without referencing performance to the HC group prior to analysis).

The optimal number of clusters was determined by evaluating agreement across 24 quantitative clustering indices (NbClust package; Charrad, Ghazzali, Boiteau, & Niknafs, 2014), with the cluster range constrained between 2 and 10. The robustness and stability of the clustering solution were evaluated through a nonparametric bootstrapping cluster analysis procedure consisting of resampling the dataset (random resampling with replacement), repeating the cluster analysis on the resampled data, computing Jaccard cluster similarity scores (representing the proportion of cases similarly clustered together in the original and resampled analysis), and repeating this procedure over 1000 iterations to evaluate the resulting overall cluster similarity scores. Cluster separation was confirmed using linear discriminant analysis. See the Supplementary Materials for additional analysis details. Similar cluster analysis approaches have successfully been used to characterize heterogeneity in CHR (Dean et al., 2018; Gupta, Cowan, et al., 2021) and schizophrenia samples (Paul et al., 2022; Strauss et al., 2013; Strauss & Herbener, 2011).

Interpretations of the cluster solution were confirmed with a 3 cluster × 4 construct mixed ANOVA. Differences in clinical and demographic profiles among cluster subgroups were then examined using one-way ANOVA or Pearson’s chi-squared tests (see Supplementary Materials for additional statistical details). Variables related to the study hypothesis of equifinality in negative symptoms were clinical group membership (CHR versus HSC) and negative symptoms (the five individual NSI-PR symptom domains and two broad dimensions; (Chang et al., 2021; Strauss, Ahmed, Young, & Kirkpatrick, 2019), with statistical significance across negative symptom variables corrected for multiple comparisons using the false-discovery rate (FDR correction; Benjamini & Hochberg, 1995). Several exploratory variables were examined to further characterize the resulting cluster subgroups. Exploratory clinical variables were positive symptoms (SIPS positive symptom total); self-reported severity of depression (CES-D; Radloff, 1977), perceived stress (PSS; S. Cohen, Kamarck, & Mermelstein, 1983), and trait anxiety (STAI; Bieling, Antony, & Swinson, 1998); social and role functioning (GF:S and GF:R; Cornblatt et al., 2007); NAPLS 1- and 2-year psychosis risk scores (CHR participants only; Cannon et al., 2016); a proxy score indexing putative primary (versus secondary; Goetz et al., 2007; Kirkpatrick, Mucci, & Galderisi, 2017; Kirschner, Aleman, & Kaiser, 2017; Tran et al., 2023) negative symptomatology (calculated as the discrepancy between negative and affective symptoms)^1^; and prevalence of DSM-5 diagnoses. Exploratory demographic variables were sex at birth, race/ethnicity, age, and education. Exploratory cognitive variables were estimated premorbid functioning (Wilkinson & Robertson, 1993), verbal learning (HVLT; Benedict, Schretlen, Groninger, & Brandt, 1998), and processing speed (BACS Symbol Coding; Keefe et al., 2004). Statistical significance for ANOVAs of exploratory variables was *p* < 0.05 uncorrected, with follow-up pairwise comparisons evaluated at *p* < 0.05 corrected for multiple comparisons across cluster groups.

Because half the participants in each group completed versions of the effort–cost computation and reinforcement learning tasks that used points instead of a monetary reward, supplemental analyses examined the influence of task version on performance across groups and across cluster subgroups (see Supplementary Materials).

## Results

### Cluster analysis

A three-cluster solution was selected, as it was favored by the plurality (11/24) of examined clustering indices, with strong support relative to the other evaluated cluster solutions (i.e., the next most-favored solution was only recommended by four clustering indices) (see Table S1 and Figure S1 of the Supplementary Materials for the clustering index results and cluster plot). The cluster-wise bootstrap analysis indicated that the three clusters were valid and highly stable (i.e., Jaccard scores ≥0.85; (Hennig, 2007, 2008, 2020) across the bootstrapped samples: cluster 1 = 0.86; cluster 2 = 0.92; cluster 3 = 0.87. Linear discriminant analysis classified cases into clusters with 96% accuracy, further supporting the separation and robustness of the cluster solution (see Table S2 of the Supplementary Material).

For the two tasks in which participants completed either the version using a points-based reward or the version using a monetary reward, the task version did not significantly influence reinforcement learning task performance. Although there was a significant overall effect of task version on effort–cost computation task performance, this effect did not significantly vary as a function of the participant group in the full sample or as a function of cluster in the cluster analysis sample (see Supplementary Materials). Therefore, the analyses retained the full participant sample.

**Figure 1.**
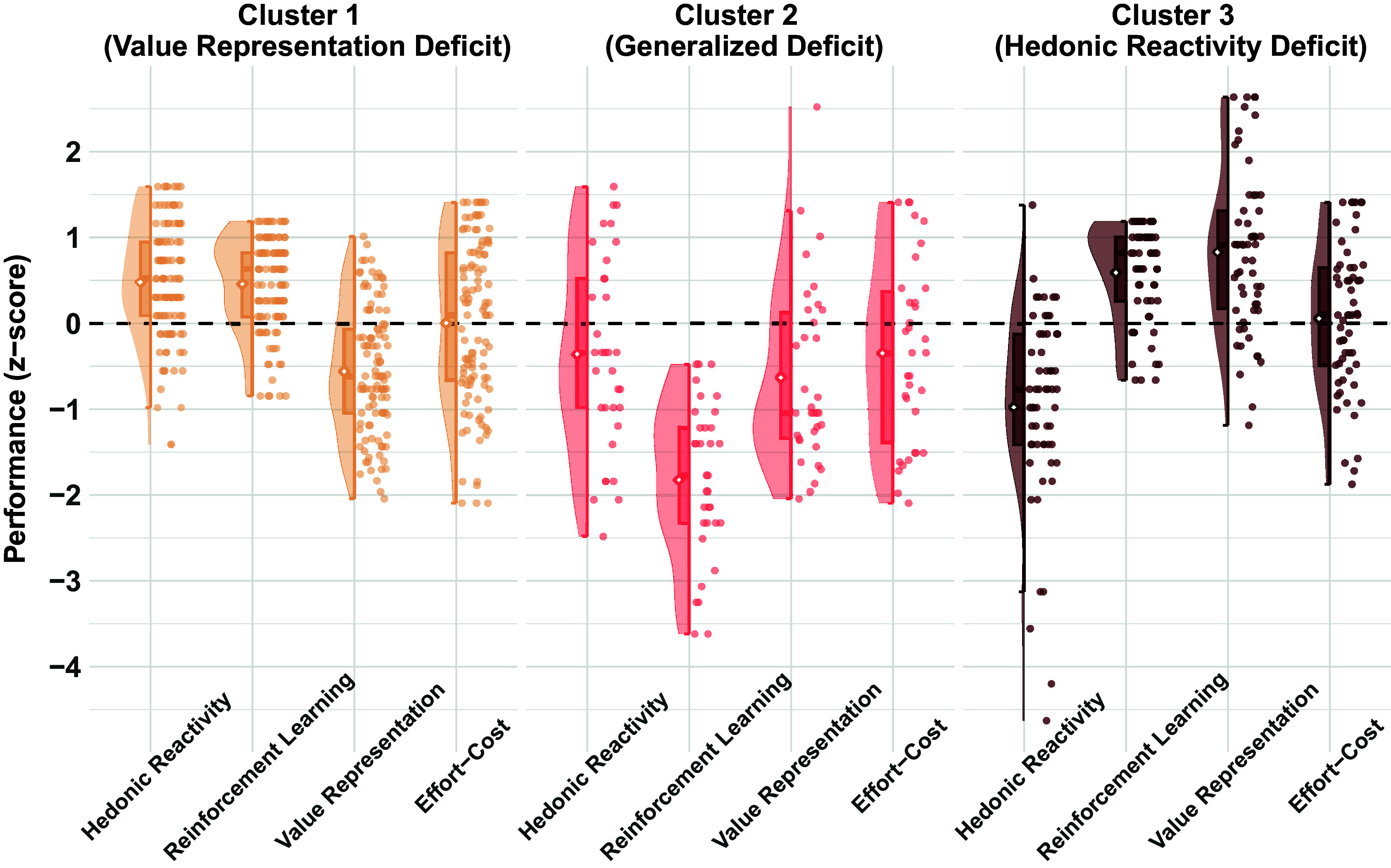
Reward processing profiles of each cluster. Clusters were characterized by a value representation deficit (cluster 1), a generalized deficit across reward processing domains (cluster 2), and a hedonic reactivity deficit (cluster 3). Diamonds denote mean scores and boxplots indicate the median and interquartile range. The dotted line at *z* = 0 represents the mean value of healthy control reference group to which task scores were *z*-scored.

Visual inspection of the cluster results (Figure 1) revealed that cluster 1 represented participants with below-average value representation but above-average hedonic reactivity and reinforcement learning (*Value Representation Deficit cluster*; *n* = 106; 54%). In contrast, Cluster 2 was composed of participants with impaired performance across all reward-processing constructs, particularly reinforcement learning and value representation (*Generalized Deficit cluster*; *n* = 34; 17%). Cluster 3 contained participants with blunted hedonic reactivity but above-average value representation and reinforcement learning (*Hedonic Reactivity Deficit cluster*; *n* = 58; 29%).

The cluster analysis was also repeated on CHR and HSC performance scores without standardizing to the HC group performance, and these results are reported in the Supplementary Material. The results between the two approaches were generally similar, both for the resulting three-cluster solution and for the pattern of cluster subgroup differences in clinical variables. However, the analysis using HC group-referenced scores led to a more stable and interpretable cluster solution, including a more clearly recommended optimal number of clusters among the evaluated cluster indices, higher cluster stability scores, and a more strongly differentiated pattern of task performance within clusters (e.g., the *Generalized Deficit cluster* had a larger magnitude of deficits across reward processing constructs). Therefore, the HC group-referenced cluster analysis results are reported here, and the full results of the cluster analysis using nonreferenced scores are reported in the Supplementary Material.

A 3-cluster × 4-construct ANOVA confirmed the cluster interpretation, which indicated a significant cluster-by-construct interaction, *F*(6585) = 50.90, *p* < 0.001, *η*_p_^2^ = 0.34, with significant cluster differences for each reward processing construct with the exception of effort–cost computation (Table 2). Follow-up comparisons indicated significantly lower value representation scores in the *Value Representation Deficit* and *Generalized Deficit* clusters compared to the *Hedonic Reactivity Deficit* cluster. In contrast, hedonic reactivity scores were significantly lower in the *Hedonic Reactivity Deficit* cluster compared to the other two clusters and significantly lower in the *Generalized Deficit* cluster compared to the *Value Representation Deficit* cluster. The *Generalized Deficit* cluster had lower reinforcement learning than the *Hedonic Reactivity Deficit* and *Value Representation Deficit* clusters. Effort–cost computation scores were below the normative HC group average and qualitatively lower in the *Generalized Deficit* cluster compared to the other two clusters; however, cluster differences were not statistically different.

**Table 2.** Cluster subgroup characteristics

	Value representation deficit (Clus1)	Generalized deficit (Clus2)	Hedonic reactivity deficit (Clus3)	Test statistic	Significant pairwise differences
*n* (%)	106 (54%)	34 (17%)	58 (29%)		
Demographic variables					
Clinical group (CHR/HSC)	61/45	19/15	30/28	*χ*^2^(2, *N* = 198) = 0.52, *p* = 0.772	–
Age	23.34 (4.42)	24.03 (4.06)	23.57 (4.39)	*F*(2,198) = 0.82, *p* = 0.443, *η*_p_^2^ = 0.01	–
Sex (M/F)	38/68	8/26	10/48	*χ*^2^(2, *N* = 198) = 6.86, *p* = 0.032	Clus1 > Clus3
Ethnicity/race				*χ*^2^(10, *N* = 197) = 11.33, *p* = 0.332	–
Asian	19.81%	23.53%	15.52%		
Black	11.32%	23.53%	8.62%		
Latinx	10.38%	5.88%	15.52%		
Multiracial	8.49%	5.88%	15.52%		
Middle Eastern	1.89%	2.94%	5.17%		
White-non-hispanic	47.17%	38.24%	39.66%		
Education (years)	14.62 (1.96)	13.97 (2.32)	14.64 (2.51)	*F*(2,190) = 1.21, *p* = 0.301, *η*_p_^2^ = 0.01	–
Clinical variables					
NSI-PR diminished motivation and pleasure factor	1.45 (0.79)	1.86 (0.79)	1.38 (0.78)	*F*(2,190) = 4.32, *p*_FDR_ = 0.028, *η*_p_^2^ = 0.04	Clus2 > Clus1 (*p* = 0.030) Clus2 > Clus3 (*p* = 0.018)
NSI-PR diminished expression factor	0.80 (0.90)	1.48 (1.24)	0.70 (0.92)	*F*_W_(2,74.8) = 5.18, *p*_FDR_ = 0.028, *η*_p_^2^ = 0.08	Clus2 > Clus1 (*p* = 0.014) Clus2 > Clus3 (*p* = 0.007)
NSI-PR anhedonia	1.15 (1.01)	1.56 (1.10)	1.14 (1.00)	*F*(2,189) = 2.25, *p*_FDR_ = 0.127, *η*_p_^2^ = 0.02	–
NSI-PR asociality	1.66 (0.94)	2.14 (1.05)	1.53 (1.00)	*F*(2,190) = 4.22, *p*_FDR_ = 0.028, *η*_p_^2^ = 0.04	Clus2 > Clus1 (*p* = 0.045) Clus2 > Clus3 (*p* = 0.016)
NSI-PR avolition	1.44 (0.99)	1.76 (0.99)	1.38 (0.91)	*F*(2,190) = 1.74, *p*_FDR_ = 0.179, *η*_p_^2^ = 0.02	–
NSI-PR blunted affect	0.91 (1.05)	1.64 (1.37)	0.79 (1.06)	*F*(2,188) = 6.72, *p*_FDR_ = 0.014, *η*_p_^2^ = 0.07	Clus2 > Clus1 (*p* = 0.004) Clus2 > Clus3 (*p* = 0.002)
NSI-PR alogia	0.44 (0.73)	1.06 (1.30)	0.43 (0.71)	*F*_W_(2,72.0) = 3.56, *p*_FDR_ = 0.047, *η*_p_^2^ = 0.07	Clus2 > Clus1 (*p* = 0.033) Clus2 > Clus3 (*p* = 0.035)
Proxy primary negative symptom score (*z*-score)^a^	−0.12 (0.95)	0.41 (1.05)	−0.03 (1.01)	*F*(2,188) = 3.63, *p* = 0.028, *η*_p_^2^ = 0.04	Clus2 > Clus1 (*p* = 0.024) Clus2 > Clus3 (*p* = 0.135; *p*_uncorrected_ = 0.045)
SIPS positive (Total)	7.75 (4.42)	7.88 (4.70)	7.64 (4.59)	*F*(2,190) = 0.03, *p* = 0.972, *η*_p_^2^ = 0.00	–
CES-D	17.12 (9.56)	19.76 (10.17)	17.65 (8.01)	*F*(2,193) = 1.05, *p* = 0.352, *η*_p_^2^ = 0.01	–
PSS	28.80 (8.90)	30.76 (9.76)	28.44 (8.19)	*F*(2,193) = 0.82, *p* = 0.444, *η*_p_^2^ = 0.01	–
STAI (Trait)	16.55 (5.62)	17.18 (6.35)	15.96 (5.29)	*F*(2,193) = 0.50, *p* = 0.606, *η*_p_^2^ = 0.01	–
Any DSM–5 diagnosis (%)	70.75%	82.35%	72.41%	*χ*^2^(2, *N* = 193) = 1.79, *p* = 0.408	–
Bipolar disorders	4.72%	11.76%	8.62%	*χ*^2^(2, *N* = 198) = 2.25, *p* = 0.325	–
Depressive disorders	31.13%	26.47%	32.76%	*χ*^2^(2, *N* = 198) = 0.41, *p* = 0.815	–
Substance use disorders	16.04%	23.53%	18.97%	*χ*^2^(2, *N* = 198) = 1.01, *p* = 0.605	–
Anxiety disorders	57.55%	55.88%	62.07%	*χ*^2^(2, *N* = 198) = 0.44, *p* = 0.803	–
Obsessive–compulsive disorders	9.43%	11.76%	6.90%	*χ*^2^(2, *N* = 198) = 1.45, *p* = 0.484	–
Trauma disorders	22.64%	32.35%	22.41%	*χ*^2^(2, *N* = 198) = 1.86, *p* = 0.395	–
Global functioning: Social	7.64 (1.25)	6.75 (1.50)	7.87 (1.28)	*F*(2,187) = 7.96, *p* < 0.001, *η*_p_^2^ = 0.08	Clus2 < Clus1 (*p* = 0.003) Clus2 < Clus3 (*p* < 0.001)
Global functioning: Role	7.96 (1.42)	7.47 (1.41)	8.18 (1.23)	*F*(2,187) = 2.77, *p* = .065, *η*_p_^2^ = 0.03	–
NAPLS 1-year psychosis conversion risk probability	8.00% (3.87)	11.90% (7.74)	8.25% (5.39)	*F*(2,89) = 3.64, *p* = 0.030, *η*_p_^2^ = 0.08	Clus2 > Clus1 (*p* = 0.030) Clus2 > Clus3 (*p* = 0.086; *p*_uncorrected_ = 0.029)
NAPLS 2-year psychosis conversion risk probability	10.60% (5.01)	15.50% (9.80)	10.90% (6.95)	*F*(2,89) = 3.54, *p* = 0.033, *η*_p_^2^ = 0.07	Clus2 > Clus1 (*p* = 0.033) Clus2 > Clus3 (*p* = 0.090; *p*_uncorrected_ = 0.030)
Current antipsychotic use	1.92%	6.25%	5.26%	*χ*^2^(2, *N* = 193) = 1.93, *p* = 0.381	–
Cognitive variables					
Estimated premorbid functioning (WRAT–4 Word Reading)	110.93 (12.17)	106.34 (15.44)	114.12 (13.90)	*F*(2,160) = 3.12, *p* = 0.047, *η*_p_^2^ = 0.04	Clus2 < Clus3 (*p* = 0.041)
Verbal learning (HVLT) Total score	27.36 (4.52)	25.00 (5.50)	27.72 (5.04)	*F*(2,186) = 3.59, *p* = 0.030, *η*_p_^2^ = 0.04	Clus2 < Clus3 (*p* = 0.038)
Verbal learning (HVLT) Learning rate (Trial 3–Trial 1)	2.63 (1.63)	3.22 (1.75)	2.67 (1.94)	*F*(2,186) = 1.46, *p* = 0.231, *η*_p_^2^ = 0.02	–
Processing speed (BACS symbol coding)	58.70 (13.70)	57.72 (11.53)	59.86 (11.38)	*F*(2,170) = 0.28, *p* = 0.758, *η*_p_^2^ = 0.00	–
Task performance					
Hedonic reactivity (average IAPS pleasantness rating)	0.48 (0.68)	−0.36 (1.13)	−0.97 (1.13)	*F*w(2,69.6) = 44.01, *p* < 0.001, *η*_p_^2^ = 0.33	Clus2 < Clus1 (*p* < 0.001) Clus3 < Clus1 (*p* < 0.001) Clus3 < Clus2 (*p* = 0.036)
Value representation (average *k*-delay discounting rate)	−0.56 (0.74)	−0.63 (1.05)	0.83 (0.94)	*F*(2,195) = 54.62, *p* < 0.001, *η*_p_^2^ = 0.36	Clus1 < Clus3 (*p* < 0.001) Clus2 < Clus3 (*p* < 0.001)
Effort–cost computation (EEfRT % hard task choices^b^)	0.01 (0.95)	−0.35 (1.07)	0.06 (0.85)	*F*(2,195) = 2.21, *p* = 0.113, *η*_p_^2^ = 0.02	–
Reinforcement learning (PRLT % Gain condition accuracy^c^)	0.46 (0.55)	−1.82 (0.90)	0.59 (0.57)	*F*w(2,74.6) = 104.19, *p* < 0.001, *η*_p_^2^ = 0.67	Clus2 < Clus1 (*p* < 0.001) Clus2 < Clus3 (*p* < 0.001)

*Note*: Values reflect mean (standard deviation) unless otherwise indicated. *p*-values with false-discover rate (FDR) correction for the seven negative symptom variables are indicated by *p*_FDR_ in the test statistic column, while unadjusted *p-*values are reported for exploratory variables. All significant pairwise comparison *p*-values for both hypothesis-related and exploratory variables are corrected for multiple comparisons unless otherwise indicated by *p*_uncorrected_.

BACS = Brief Assessment of Cognition in Schizophrenia; CHR = clinical high-risk participants; CES-D = Center for Epidemiologic Studies – Depression Scale; EEfRT = effort expenditure for rewards task; *F*_w_ = Welch’s *F*-statistic; HSC = help-seeking control participants; NSI-PR = negative symptom inventory – psychosis risk; PSS = Perceived Stress Scale; SIPS = structured interview for psychosis-risk syndromes; STAI = State-Trait Anxiety Inventory; WRAT-4 = Wide-Range Achievement Test – 4; HVLT = Hopkins Verbal Learning Test; IAPS = International Affective Picture System; PRLT = probabilistic reinforcement learning task; *p*_FDR_ = false-discovery rate-corrected *p*-value.

a Proxy primary negative symptom score is the scaled difference score of N1 + N2 + N3 - G2 - G4 SIPS items.

b Percentage of hard task choices for trials in the upper half of the reward magnitude range and in the upper (50%–88%) reward probability range.

c Percentage of correct choices on the most frequently rewarded gain stimulus pairing across learning trials.

### Cluster comparisons of external variables

Clinical and demographic characteristics of each cluster subgroup and their statistical comparisons are reported in Table 2.

#### Variables related to testing the hypothesis of equifinality in negative symptoms

**Figure 2.**
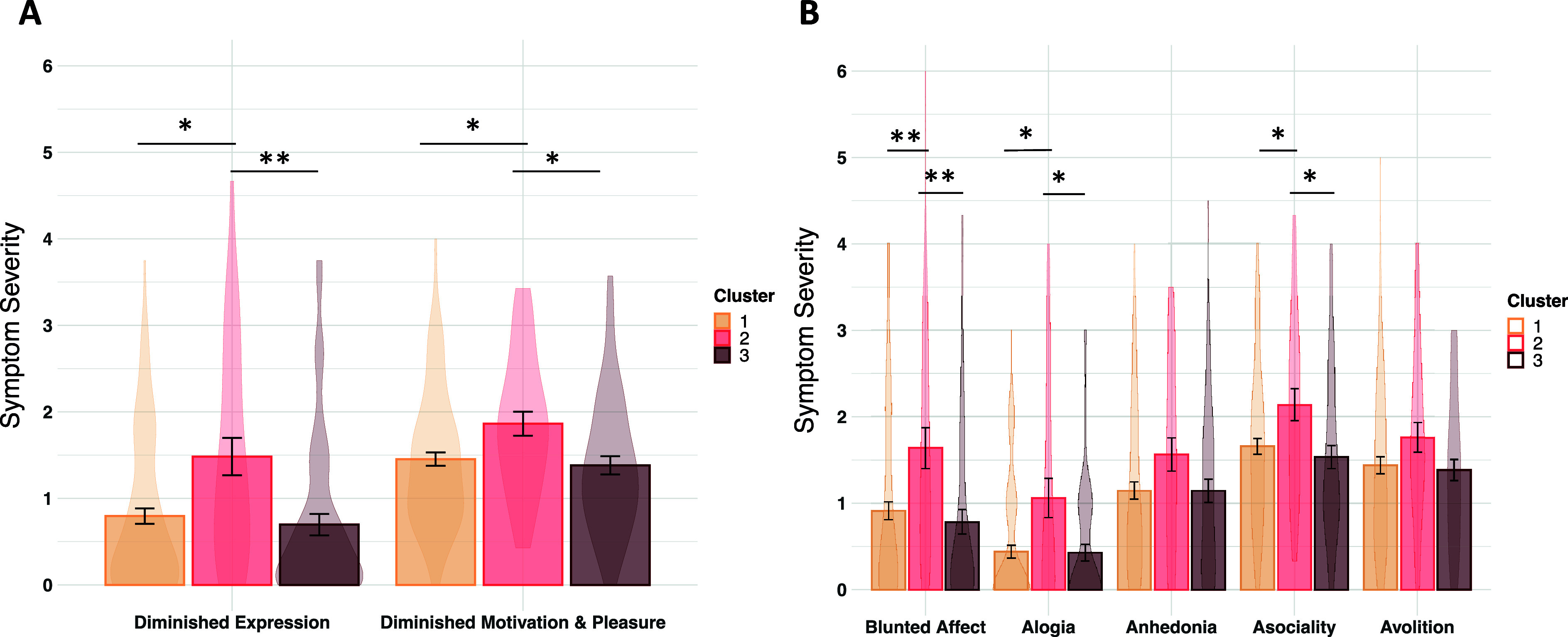
Negative symptom severity in each cluster. (a) Mean negative symptom factor scores across clusters. (b) Mean negative symptom domain scores across clusters. * *p* < 0.05. ** *p* < 0.01.

Clusters did not significantly differ in the proportion of CHR and HSC participants. Comparing clusters on negative symptoms indicated greater symptom severity in the *Generalized Deficit* cluster compared to the other two clusters, for both the diminished motivation and pleasure and the diminished expression symptom factors (FDR-corrected *p* < 0. 05) (Figure 2). Cluster differences were also found (FDR-corrected *p* < 0. 05) when examining the five individual negative symptom domains, with more severe asociality, blunted affect, and alogia in the *Generalized Deficit* cluster compared to the other two clusters (Figure 2). Cluster comparisons on negative symptoms were also performed in the CHR group only (evaluated using uncorrected *p* < 0.05 due to the reduced sample size), with consistent results for all negative symptom factors and domains except for alogia, which was no longer significantly different between clusters (see Table S4 of the Supplementary Material).

Despite the cluster differences in negative symptom severity, there was nonetheless some indication that clinically significant negative symptoms were present across the three reward processing clusters when examining the proportion of participants with mild-or-greater (rating of ≥2) item-level symptom severity ratings, particularly for the motivation and pleasure factor and the asociality domain where proportions were above 50% (see Table S3 of the Supplementary Material), and when examining negative symptom severity in CHR participants within clusters (see Table S4 of the Supplementary Material).

#### Exploratory variables to characterize differences across clusters

When comparing clusters on exploratory external variables, clusters were comparable in terms of most demographic variables (with the exception of a greater proportion of females in the *Hedonic Reactivity Deficit* compared to the *Value Representation Deficit* cluster), self-reported symptom severity, severity of subthreshold psychotic symptoms, and rates of current psychiatric diagnoses. The *Generalized Deficit* cluster had reduced social functioning, greater proxy primary negative symptom scores (reflecting greater likelihood of primary negative symptoms; see footnote), higher psychosis conversion risk probability scores, lower estimated premorbid intellectual functioning, and reduced total verbal learning performance.

## Discussion

The current study aimed to identify data-driven reward processing profiles and their associated negative symptom presentation in a transdiagnostic clinical help-seeking sample. Cluster analysis of CHR and HSC participant performance across four reward processing constructs previously associated with negative symptoms (hedonic reactivity, reinforcement learning, value representation, effort–cost computation) revealed three distinct clusters. Clusters were primarily characterized by: (1) a value representation deficit, (2) a generalized reward processing deficit, or (3) a hedonic reactivity deficit.

As hypothesized, clusters did not differ in rates of clinical group membership (CHR versus HSC) or psychiatric diagnoses, suggesting that distinct reward processing profiles are transdiagnostic and not bound by diagnostic categories. In contrast, the hypothesized presence of multiple mechanistic pathways to negative symptom presentation (i.e., equifinality) was not strongly supported. Negative symptoms were most robustly observed and at greatest severity in the *Generalized Deficit* cluster compared to the clusters where deficits were restricted to a specific reward processing component, suggesting that negative symptoms were associated with a single, more global reward processing deficit.

Although an equifinality perspective has been proposed to explain the transdiagnostic nature of negative symptoms across different psychiatric disorders (Nusslock & Alloy, 2017; Strauss & Cohen, 2017; Whitton et al., 2015), few prior studies have included multiple clinical groups, assessed more than one reward processing construct, or targeted heterogeneous CHR and HSC samples that capture more normative variation in negative symptom presentation and reward system functioning than those of traditional studies restricted to individuals with depression and/or schizophrenia. The current results suggest that heterogeneity in reward-processing alterations is transdiagnostic and exists *within* diagnostic categories; however, a single global reward-processing deficit profile has the strongest association with negative symptom severity. These results build on prior studies that have documented multiple reward processing alterations in CHR samples, albeit studied individually (Gruber et al., 2018; Millman et al., 2020; Pratt, Treadway, Strauss, & Mittal, 2024; Strauss et al., 2023; Strauss, Ruiz, et al., 2018), and help to explain prior findings of heterogeneous overlap and divergence in reward processing dysfunction relative to other psychiatric diagnoses like schizophrenia and depression. Furthermore, the current finding of distinct profiles of reward processing dysfunction but with only the *Generalized Deficit* cluster clearly showing elevated negative symptom severity is consistent with a recent study (Luther et al., 2024) using a similar data-driven approach that identified a single, global reward processing deficit underlying negative symptoms in a transphasic serious mental illness sample (i.e., including participants across putative phases of psychotic illness). These findings suggest that in contrast to the equifinality model (Nusslock & Alloy, 2017; Whitton et al., 2015) in which different mechanistic pathways between disorders (e.g., schizophrenia versus depression) can give rise to the common presence of negative symptoms, a single, generalized reward-processing deficit was most strongly associated with negative symptoms when examined in a transdiagnostic sample of help-seeking and CHR participants. It may be that equifinality is not as strongly supported within a sample of help-seeking and CHR youth, where symptoms may be present in milder form and where psychopathological processes might still be emerging through developmental and/or environmental factors. Nonetheless, there was some indication for the presence of mild negative symptoms across clusters, particularly asociality and the motivation and pleasure factor. Future research is needed to corroborate the presence and nature of these findings; for example, whether the symptoms observed in the other clusters reflect secondary negative symptoms, as suggested by the greater estimated primary negative symptom score in the *Generalized Deficit* cluster.

In contrast to the *Hedonic Reactivity Deficit* and *Value Representation Deficit* clusters, where below-average performance was only observed in a single reward-processing construct relative to the normative group, the *Generalized Deficit* cluster was characterized by deficits across the four reward-processing constructs. These findings suggest that a more global reward-processing deficit profile is the predominant pathway to negative symptom formation. However, it should be noted that reinforcement learning, followed by value representation, were the most prominent deficits in this cluster. Prior research in schizophrenia samples indicates that difficulties representing the expected value of rewards during decision-making contribute to reward learning deficits, particularly in participants with elevated negative symptoms (Gold et al., 2012; Gold, Waltz, Prentice, Morris, & Heerey, 2008; Hernaus et al., 2019; Hernaus, Gold, Waltz, & Frank, 2018; Strauss & Herbener, 2011; Waltz, Frank, Robinson, & Gold, 2007; Waltz, Frank, Wiecki, & Gold, 2011). Therefore, an alternate possibility is that a reward processing profile predominantly consisting of reinforcement learning deficits characterized by value representation abnormalities may be a particularly strong pathway toward negative symptom formation. The *Value Representation Deficit* cluster did not clearly display elevated negative symptoms, further suggesting that a value representation deficit may be most relevant to negative symptom formation when this deficit interacts with or contributes to other reward processing difficulties (e.g., when value representations cannot be updated efficiently to guide learning and decision-making; Gold et al., 2008). Additional research is needed to further characterize the potential additive and/or interactive effects of multiple reward processing deficits on negative symptom severity in the context of equifinality.

In addition to having the greatest severity of negative symptoms, the *Generalized Deficit* cluster also had reduced social functioning, greater proxy scores of putative primary (versus secondary) negative symptoms, as well as the highest psychosis conversion risk probability scores. This cluster nonetheless had comparable severity of most other symptoms and several measures of cognition, suggesting that this clinical profile was not simply a correlate of greater overall symptom load. However, this cluster demonstrated reduced estimated premorbid intellectual functioning and overall verbal learning performance (but not rate of learning or processing speed), suggesting the potential contribution of subtle cognitive deficits to the observed reward processing deficits (e.g., reinforcement learning). Taken together, these results suggest that a global reward-processing deficit profile is associated with increased functional impairment and confers greater psychosis risk. These findings are consistent with longitudinal research indicating that more elevated baseline negative symptoms predict transition to psychosis in CHR youth (Gupta et al., 2021; Piskulic et al., 2012) and further suggest it may be possible to identify a reward-processing profile associated with increased psychosis risk. Task-based assessment of reward processing profiles might therefore provide an objective and scalable measure of psychosis risk-related mechanisms to enhance the sensitivity and precision of existing risk calculators (Cannon et al., 2016; Zhang et al., 2019). Planned longitudinal analysis of the ongoing CAPR study will address this aim.

Another finding was the greater proportion of females in the *Hedonic Reactivity Deficit* cluster compared to the *Value Representation Deficit* cluster. Given that there was some indication for clinically significant negative symptoms present across the three reward processing clusters, and that the *Hedonic Reactivity Deficit* cluster had the largest deficit in hedonic reactivity compared to the other two clusters, it is possible that a hedonic reactivity deficit pathway to negative symptoms may be more prominent in females than males. However, it should be noted that there were no significant differences in the proportion of females between the *Hedonic Reactivity Deficit* and *Generalized Deficit* clusters. Hedonic reactivity deficits are also reported in individuals with depressive disorders (Bylsma et al., 2008), where reward responsiveness is associated with symptoms of anhedonia (Pizzagalli, Iosifescu, Hallett, Ratner, & Fava, 2008), and where there is a greater prevalence of depression in women compared to men (Salk, Hyde, & Abramson, 2017). However, there were no cluster differences in anhedonia severity or rates of depressive disorders. Therefore, further research is needed to examine the potential presence of sex-related differences in hedonic reactivity in the context of negative symptoms transdiagnostically.

An outstanding question concerns the neurobiological correlates of the identified performance-based reward-processing profiles. Although prior research has documented alterations in frontal–striatal systems during reward processing in psychosis (Barch & Dowd, 2010; Chase, Loriemi, Wensing, Eickhoff, & Nickl-Jockschat, 2018; Kesby, Murray, & Knolle, 2023; Radua et al., 2015), depression (Keren et al., 2018), and in CHR youth (Howes, Hird, Adams, Corlett, & McGuire, 2020; Kesby et al., 2023; Radua et al., 2015), future neuroimaging research that leverages heterogeneity in symptom presentation independent of clinical diagnosis and focuses on cross-task performance patterns will help to address this question. Similarly, examining whether performance-based reward processing profiles map onto subgroups identified in prior biotype-based stratification studies (Clementz et al., 2020; Dinga et al., 2019; Drysdale et al., 2017; Dwyer et al., 2022; Planchuelo-Gómez et al., 2020) may help clarify neurobiological underpinnings of distinct reward processing profiles with the potential to aid in negative symptom treatment innovation.

Although clusters were characterized by patterns of reward processing deficits relative to the HC group serving as the reference sample, there were also areas of greater reward processing performance relative to the HC group. Specifically, the *Value Representation Deficit* cluster had above-average hedonic reactivity and reinforcement learning, while the *Hedonic Reactivity Deficit* cluster had greater value representation and reinforcement learning. The above-average reinforcement learning in these clusters is consistent with the interpretation that the reinforcement learning deficit unique to the *Generalized Deficit* cluster is an important feature of the elevated negative symptom presentation in this cluster. Additionally, the opposite profiles of hedonic reactivity and value representation in the *Hedonic Reactivity Deficit* and *Value Representation Deficit* clusters suggest a reciprocal relationship: participants with elevated in-the-moment hedonic reactivity were more prone to discounting the value of a delayed reward (due to difficulty maintaining a representation of the value of the delayed reward), while participants with blunted hedonic reactivity were more likely to display elevated value representation (due to being less likely to discount the value of the delayed reward). However, given that deficits in both hedonic reactivity and value representation were found in the *Generalized Deficit* cluster, the relationship between these two constructs may depend on the extent to which there is co-occurring dysfunction in other aspects of reward processing (e.g., in reinforcement learning and effort–cost computation). Given that the observed reward processing profiles were reproduced when the cluster analysis was performed using the unreferenced task scores (i.e., when task scores were scaled within the clinical sample rather than scaled relative to the HC group; see the Supplementary Material), the pattern of elevations and deficits in reward processing performance across clusters was not attributable to the HC group’s reward processing task performance. However, the *Generalized Deficit* cluster was the cluster that most clearly displayed elevated negative symptoms and had significant findings on other examined clinical variables (e.g., social functioning, psychosis risk conversion probability); therefore, the clinical significance of the higher performance scores relative to the HC group in the *Hedonic Reactivity Deficit* and *Value Representation Deficit* clusters may be limited.

Similarly, in the full sample, none of the help-seeking participant groups (i.e., CHR or HSC) displayed significantly reduced task performance compared to the HC group, and the CHR group had comparable performance to HSC peers (Table 1). Prior studies have documented reward processing abnormalities in CHR samples relative to community control groups; however, findings have been mixed across studies and constructs examined (Ermakova et al., 2018; Gruber et al., 2018; Karcher, Hua, & Kerns, 2019; Millman et al., 2020; Strauss et al., 2023; Strauss, Ruiz, et al., 2018). Furthermore, recent meta-analytic work on neuropsychological deficits in CHR samples has indicated that any excess impairment in CHR groups relative to HSCs is predominantly attributed to CHR participants who transition to psychosis, with comparable deficits in CHR participants without transition to psychosis (Millman et al., 2022). Similar to the meta-analytic findings regarding neuropsychological profiles, and consistent with the current cluster analysis results of more global reward processing deficits and reduced neuropsychological functioning in the cluster of participants with the greatest psychosis conversion risk probability scores, the heterogeneous reward processing profiles in youth at CHR might not be fully represented when analyzed at the group level. These findings highlight the importance of accounting for clinical heterogeneity in the study of psychosis risk, such as by using data-driven stratification approaches like the cluster analysis in the current study.

The study has several limitations. First, although the observed cluster solution was found to be stable and robust when evaluated against bootstrapped samples, the participant sample size was small for data-driven analysis and results warrant validation in external samples. Second, the study was cross-sectional, preventing firm conclusions about the causal contributions of the observed reward processing profiles to the development of negative symptoms. Planned longitudinal follow-up assessment of CAPR study participants will help clarify the stability of reward processing profiles and determine whether these represent mechanistic pathways to symptom development or common consequences of shared etiological factors. Third, negative symptom severity was generally mild across clinical groups and additional research is needed to determine whether the identified transdiagnostic reward processing profiles generalize to samples with more severe negative symptoms. However, negative symptom severity in the current sample was comparable to that of previously published CHR samples (e.g., Chang et al., 2021; Gupta, Strauss et al., 2023), Furthermore, mild negative symptom severity scores are typically observed even in schizophrenia samples when not specifically selecting for participants with severe negative symptoms or deficit syndrome presentation (e.g., Strauss, Nunez, et al., 2018 Supplement). Fourth, the assessment of reward profiles was limited to four component processes; future studies using an expanded array of RDoC reward-processing subconstructs (e.g., reward anticipation) will provide a more comprehensive characterization of profiles associated with negative symptoms. Fifth, multiple processes contribute to the probabilistic reinforcement learning construct, including hedonic reactivity, value representation, and other sub-processes (e.g., prediction error signaling), which will require further analysis to uncover the origins of this deficit (Gold et al., 2012).

In conclusion, this study indicates that distinct data-driven reward processing profiles can be identified transdiagnostically but that negative symptoms are most strongly linked to a single profile of global reward processing deficits. These findings support the view that reward processing dysfunction is heterogeneous both between and within psychiatric disorders but do not support a transdiagnostic equifinality model of negative symptoms, where both shared and distinct pathways to negative symptoms can be present across and within psychiatric disorders. Future studies are needed to determine whether computerized assessment of reward-processing constructs can assist in the development of individualized prediction and treatment of negative symptoms.

## Supporting information

Spilka et al. supplementary material Spilka et al. supplementary material

## Footnotes

1 Proxy scores for primary negative symptoms (versus negative symptoms secondary to other factors such as co-occurring depression) were computed from SIPS ratings, modeled after the approaches of Kirkpatrick, Mucci, & Galderisi, 2017 and Goetz et al., 2007 for the identification of deficit syndrome in schizophrenia based on the difference in severity of core negative symptoms versus mood and anxiety symptoms. Proxy scores were calculated from the sum of social anhedonia, avolition, and emotional expression SIPS ratings (N1 + N2 + N3) subtracted by the sum of dysphoric mood and stress intolerance SIPS ratings (G2 + G4), with the obtained difference score scaled across the combined sample of CHR and HSC participants. Similar approaches have been used to characterize negative symptom presentation in CHR samples (Tran et al., 2023).

## Supplementary material

The supplementary material for this article can be found at http://doi.org/10.1017/S003329172400326X.
