# Supplementary material for: A generalized reward processing deficit pathway to negative symptoms across diagnostic boundaries
Source: Psychol Med. 2025 Feb 4;55:e6. doi: 10.1017/S003329172400326X (PMC11968125; doi:10.1017/S003329172400326X)
Supplement: Spilka et al. supplementary material [file S003329172400326Xsup001.docx]

**Supplementary Material**

**Methods**

***Participant recruitment and testing procedures***

CHR and HSC participants were recruited through targeted online and print advertisements, email, and through contact with mental health providers in hospital, community mental health center, and school settings. CHR and HSC participants were excluded if they had a lifetime history of psychotic disorder. CHR and HSC participants were allowed to have a current alcohol or cannabis use disorder of mild to moderate severity, as long as psychosis-risk symptoms did not occur exclusively during periods of intoxication in the case of CHR participants. Participants were excluded if they met criteria for a severe current alcohol or cannabis use disorder or met criteria for other current substance use disorders (hallucinogen, opioid, stimulant) of any severity.

HSC participants were participants who were referred/self-referred for psychosis-risk symptoms and/or had at least one current DSM-5 non-psychotic disorder diagnosis but did not meet criteria for a SIPS psychosis-risk syndrome. For example, an HSC participant may have been an individual who was referred to a study site’s clinical high-risk research program (e.g., the University of Georgia Psychology Department’s Georgia Psychiatric Risk Evaluation Program; G-PREP) by a clinician in the community for the purpose of performing a diagnostic assessment related to emerging psychotic experiences, found to not meet CHR criteria but meet diagnostic criteria for a DSM-5 non-psychotic disorder diagnosis, and then recruited into the study.

HC participants were recruited through online and print advertisements, and email. HC participants were excluded if they met criteria for any lifetime DSM-5 diagnosis, SIPS psychosis risk syndrome, antipsychotic medication use, or had a family history of psychosis.

Additional exclusion criteria for all participants were lifetime history of neurological disorder or brain injury.

Participation occurred through one of eight study sites: University of California Irvine, University of Maryland Baltimore, University of Maryland Baltimore County, Northwestern University, Temple University, University of Georgia, Emory University, and Yale University. The study procedure was the same at each site. Participants underwent an initial phone screening to determine general eligibility for the study. A series of clinical interviews, questionnaires (adapted to online format), cognitive tests, and computerized experimental tasks were then completed online via videoconference (due to COVID-19 pandemic-related safety policies) and administered by trained research staff. Participants were guided through the tasks by research staff who monitored progress of participants’ questionnaire and task completion in real time via screen share. Pilot testing was performed to ensure the feasibility or remote participant testing and validity of the obtained task data prior to the study start.

All participants provided verbal informed consent prior to phone screening and written consent prior to clinical interviews. Participants who were minors at the time of recruitment provided assent and required consent by a parent or legal guardian prior to participation. The study protocol was approved by Northwestern University’s Institutional Review Board (IRB), which served as the IRB of record for the study.

***Clinical measures***

Eligibility and group membership were confirmed via the SIPS and the Structured Clinical Interview for DSM-5 (First et al., 2015). Additional measures were administered to assess symptom severity, global functioning, and clinical history. Clinical interviews relevant to the current investigation were the Negative Symptom Inventory – Psychosis Risk (Strauss et al., 2023) (CHR and HSC only), the Global Functioning: Social and Role Scales (Cornblatt et al., 2007), and the Family Interview for Genetic Studies (Maxwell, 1992) and Childhood Trauma and Abuse Scale (Janssen et al., 2004) for use in the calculation of a NAPLS psychosis risk score (Cannon et al., 2016).

Self-report symptom measures included the Center for Epidemiological Studies Depression Scale (CES-D; Radloff, 1977), Perceived Stress Scale (PSS; Cohen et al., 1983), and State-Trait Anxiety Inventory Trait form (modified to include only items that load onto anxiety; Bieling et al., 1998). Cognitive tests were the Wide-Range Achievement Test – 4 (WRAT-4; Wilkinson & Robertson, 1993) Word Reading subtest to estimate premorbid IQ, and the Hopkins Verbal Learning Test – Revised (HVLT-R; Benedict et al., 1998) and Brief Assessment of Cognition in Schizophrenia (BACS; Keefe et al., 2004) Symbol Coding subtest needed to calculate a NAPLS psychosis risk score.

***Experimental tasks***

*Hedonic Reactivity*

Hedonic reactivity was assessed using a Hedonic Reactivity Task in which participants were presented with a subset of pleasant, unpleasant, and neutral photographs from the International Affective Picture System (IAPS; Lang et al., 1997), and provided self-report ratings about their level of positive emotion, negative emotion, and level of arousal/excitement in response to each photograph. There were 72 photographs (24 positive, 24 negative, and 24 neutral). Each stimulus was presented with a visual unipolar scale from 1 (not at all) to 5 (extremely) that also indicated the current self-report rating required. The task was self-paced such that a participant response triggered the appearance of the next rating scale within a given trial or the next trial following the final rating of the current trial.

The dependent variable for the current analysis was each participant’s average positive valence rating across pleasant stimulus trials.

*Reinforcement Learning*

Reinforcement learning was assessed using the Probabilistic Reinforcement Learning Task (PRLT; Gold et al., 2012), an explicit reinforcement learning task assessing learning from gains and losses. During an initial learning phase, participants were presented with 4 different stimulus choice pairs over 160 trials, and received reinforcement feedback probabilistically based on each choice. Two stimulus pairs were associated with the potential gain of a reward for correct choices, with one pair leading to reward on 90% of correct choices and the other on 80% of choices, while incorrect choices did not receive a reward. The other two stimulus pairs were associated with the potential avoidance of a loss (either 90% or 80% probability of loss avoidance), while incorrect choices led to loss. Participants were randomized to complete a version of the task using either monetary (5¢) or points (5 points) as the reward/loss outcome, and participants completing the monetary reward version received 100% of their earnings as bonus study remuneration. Each stimulus pair was presented 40 times in a randomized order during the learning phase. The learning phase was followed by a transfer phase that included trials of original and novel stimulus pairings (not analyzed here). Stimuli were pairs of landscape images, presented one at a time until participants made a choice, which triggered a feedback screen: “+5 Win!” or “Keep your points!” for respective gain and loss rewarded correct choices, or “Not a winner, try again!” or “−5 Lose!” for incorrect or non-rewarded gain and loss trials, respectively. Participants completed 12 practice trials (6 gain pairs and 6 loss pairs) prior to starting the learning phase.

The dependent variable for the current study was the percentage of correct choices on the most frequently rewarded gain stimulus pairing across learning trials, representing overall ability to learn from positive feedback during the most explicitly rewarding condition. This 90% gain condition has been associated with the largest learning deficit in high-negative symptom individuals with schizophrenia (Gold et al., 2012).

*Value Representation*

Value representation is commonly assessed through delay discounting, which indexes the representation of the subjective value of a future reward as a function of its magnitude and delay in delivery. On the Delay Discounting Task (Kirby et al., 1999), participants completed 27 trials in which they made a hypothetical choice between receiving a smaller immediate reward and a larger reward available after a delay (7-186 days). Reward size for each choice varied across trials, with the smaller immediate reward including small ($11–34), medium ($20–54), and large ($31–80) rewards, and the larger delayed reward including small ($25–35), medium ($50–60), and large ($75–85) reward values.

The dependent variable of interest was the participant’s average *k*-discounting rate across trials, estimated independently at each reward magnitude using a hyperbolic discounting model (see Gray et al., 2016; Kirby et al., 1999). This *k*-discounting rate represents the degree to which a delay in reward delivery discounts the value of the reward. The discounting rate was averaged across reward sizes and log-transformed (Gray et al., 2016), such that less negative values indicate greater discounting rates.

*Effort-Cost Computation*

The effort-cost computation paradigm in the current study was the Effort Expenditure for Rewards Task (EEfRT; Treadway et al., 2009). On each trial, participants had to chose between completing a lower effort task (“Easy Task”: 30 key presses within 7s using the dominant hand index finger) for a smaller reward (100 points) or a higher effort task (“Hard Task”: 100 key presses within 21s using the non-dominant hand little finger) for a larger reward (124-430 points). The probability of receiving the reward upon successful trial completion was also manipulated and specified during the choice period, which applied to both the Easy and Hard tasks: either 12% (low), 50% (medium), or 88% (high). These different reward probabilities were evenly distributed across task trials. Participants were randomized to complete a version of the task using either points or money (1¢ per point) as the reward, and participants completing the monetary reward version received 10% of their task earnings as bonus study remuneration. Each trial began with a 5 s choice period displaying the two task choices including the reward magnitude and probability of obtaining a reward, and the task was randomly selected if the participant did not make a choice within the 5s window. A 1s preparation screen displaying the word “Ready?” was then presented, followed by the performance period in which participants completed their selected task while a progress bar indicated progress toward task completion. Once the performance period elapsed, a feedback screen indicated whether a reward was provided. Participants were instructed to complete as many trials as possible within 20 minutes. Participants completed 4 practice trials prior to beginning the task.

The dependent variable for the current study was the percentage of hard task choices for trials in the upper half of the reward magnitude range (277-430 points) and where the probability of receiving a reward was 50% or 88%. Prior research in schizophrenia indicates group differences are largest for greater reward magnitudes and higher reward probabilities (Culbreth et al., 2018).

***Data processing***

Data processing and analysis were performed in R version 3.6.3 using RStudio 1.4.1106 (R Core Team, 2020) with the following packages: cluster, ggpubr, fpc, irr, MASS, NbClust, rstatix, stats, tidyverse.

For the hedonic reactivity task, one CHR participant was flagged for exclusion due to providing the same rating across all task trials (for positive, neutral, and negative images).

For the value representation task, the average *k-*discounting rate was estimated independently at each reward magnitude by fitting the participant’s choice indifference point to a hyperbolic discounting model (see Gray et al., 2016; Kirby et al., 1999), and then averaged to provide an overall *k*-discounting rate. Following recommended practices (Gray et al., 2016), response consistency rates were calculated to identify and exclude participants with unreliable *k*-discounting values. Inconsistency was defined *a priori* as <70% (fewer than 6/9 consistent trial responses) at any reward magnitude, leading to seven participants (4 CHR, 1 HSC, 1 HC) being flagged for exclusion.

For the Effort Expenditure for Rewards Task, trials in which participants did not make a choice within the 5 s choice window were excluded. The mean number of timed-out choice trials was 1.48 for CHR, 0.64 for HSC, and 0.83 for HC participants. Eight participants with timed-out choices on over 50% of trials were identified for exclusion (3 CHR, 2 HSC, 3 HC).

For each dependent variable, participants with extreme outlier scores (exceeding 3 times the interquartile range below the 25th percentile or above the 75th percentile) were excluded (2 CHR and 2 HC participants due to extreme PRLT task scores).

Only participants with complete data across the four tasks were retained in the final sample, which included 110 CHR, 88 HSC, and 66 HC participants.

***Cluster analysis stability***

The stability of the clustering solution was evaluated through a nonparametric bootstrapping cluster analysis procedure: the data were resampled with replacement, the cluster analysis was repeated, and Jaccard similarity scores were obtained by comparing resampled data clusters to their most similar original clusters. This procedure was repeated over 1000 iterations, and the mean similarity scores for each cluster were computed over all iterations. Jaccard cluster similarity scores represent the proportion of cases from the original clusters that were clustered together in the resampled data. A mean Jaccard similarity score of > 0.75 indicates a valid, stable cluster, and a score of 0.85 or above indicates highly stable clusters (Hennig, 2007, 2008, 2020). Separation of cluster subgroups was evaluated from the classification accuracy of a linear discriminant analysis using leave-one-out cross-validation, with cluster membership as the grouping variable and task performance as input variables.

***Additional statistical analysis details***

Where relevant, ANOVAs used Welch’s *F* when the homogeneity of variance assumption was violated, and Greenhouse-Geisser adjustments to degrees of freedom were made when the assumption of sphericity was violated. Statistically significant findings (*p* < .05, two- tailed) were followed by pairwise comparisons when relevant and corrected for multiple comparisons using the Bonferroni correction, or using the Games-Howell test for pairwise comparisons when the homogeneity of variance assumption was violated, with Tukey’s method for multiple comparisons correction.

**Results**

***Analysis comparing task versions***

*Group comparisons*

A 3 group × 2 task version ANOVA for effort-cost computation performance indicated a main effect of task version, *F*(1,258) = 15.28, *p*  < .001, η_p_^2^ = 0.06, while the main effect of group, *F*(2,258) = 1.60, *p*  = .204, η_p_^2^ = 0.01, and group by task version interaction, *F*(2,258) = 2.21, *p*  = .112, η_p_^2^ = 0.02, were not significant. Participants completing the task for monetary reward made a higher percentage of hard task choices (*M*  = 65.75%, *SD* = 25.26) than those completing the task for points (*M*  = 51.60%, *SD* = 27.80); however, this difference did not vary as a function of group.

A 3 group × 2 task version ANOVA for reward learning performance returned nonsignificant results for the main effect of task version, *F*(1,258) = 2.90, *p*  = .090, η_p_^2^ = 0.01, main effect of group, *F*(2,258) = 0.45, *p*  = .641, η_p_^2^ = 0.00, and group by task version interaction, *F*(2,258) = 0.38, *p*  = .686, η_p_^2^ = 0.00.

*Cluster comparisons*

A 3 cluster × 2 task version ANOVA for effort-cost computation performance indicated a main effect of task version, *F*(1,192) = 111.82, *p*  < .001, η_p_^2^ = 0.06, while the main effect of cluster, *F*(2,192) = 1.87, *p*  = .157, η_p_^2^ = 0.02, and cluster by task version interaction, *F*(2,192) = 1.76, *p*  = .175, η_p_^2^ = 0.02, were not significant. Participants completing the task for monetary reward overall made a higher percentage of hard task choices (*M_Z_*  = 0.27, *SD* = 0.83) than those completing the task for points (*M_Z_*  = −0.35, *SD* = 0.96); however, this difference did not vary as a function of cluster subgroup.

A 3 cluster × 2 task version ANOVA for reward learning performance indicated a main effect of cluster, *F*(2,192) = 182.13, *p*  < .001, η_p_^2^ = 0.66, while the main effect of task version, *F*(1,192) = 0.03, *p*  = .870, η_p_^2^ = 0.00, and cluster by task version interaction, *F*(2,192) = 0.16, *p*  = .850, η_p_^2^ = 0.00, were not significant.

***Table S1. Clustering index results***

| Number of clusters | Number of indices proposing this cluster solution |
| --- | --- |
| 2 | 4 |
| **3** | **11** |
| 4 | 2 |
| 5 | 0 |
| 6 | 1 |
| 7 | 1 |
| 8 | 0 |
| 9 | 2 |
| 10 | 2 |

*Note*. Twenty-four quantitative clustering indices were evaluated from the NbClust package (Charrad et al., 2014), representing all available indices in the packages other than GAP, Gamma, Gplus, and Tau, and excluding the two graphical indices (Hubert and dindex). One of the 24 examined cluster indices proposed a 1-cluster solution, which fell outside the pre-specified range of 2-10 clusters considered for analysis.

***Cluster plot***

***Figure S1.*** Cluster plot of 3-cluster solution in Principal Component Space. Ellipses represent Euclidean distance from the cluster center.

***Table S2. Linear discriminant analysis (LDA) classification table***

| Actual cluster assignment | LDA predicted assignment | | |
| --- | --- | --- | --- |
|  | Cluster 1 | Cluster 2 | Cluster 3 |
| Cluster 1 | 106 | 0 | 0 |
| Cluster 2 | 2 | 32 | 0 |
| Cluster 3 | 5 | 0 | 53 |

*Note.* Linear discriminant analysis overall classification accuracy: 96%

***Table S3. Additional negative symptom characterization of the cluster subgroups***

| Symptom factor/domain |  | Proportion of mild-or-above item-level severity^a^ within domains/factors | | | | |
| --- | --- | --- | --- | --- | --- | --- |
|  |  | Cluster 1  (Value Representation Deficit) |  | Cluster 2 (Generalized Deficit) |  | Cluster 3  (Hedonic Reactivity Deficit) |
| Diminished Motivation and Pleasure Factor |  | 0.90 |  | 0.97 |  | 0.85 |
| Diminished Expression Factor |  | 0.41 |  | 0.62 |  | 0.28 |
| Anhedonia |  | 0.44 |  | 0.62 |  | 0.48 |
| Asociality |  | 0.77 |  | 0.85 |  | 0.67 |
| Avolition |  | 0.48 |  | 0.59 |  | 0.43 |
| Blunted Affect |  | 0.40 |  | 0.62 |  | 0.28 |
| Alogia |  | 0.12 |  | 0.29 |  | 0.05 |

^a^Proportion of participants with item-level scores ≥ 2.

The possible severity range of NSI-PR symptom factor/domain scores is 0-5.

***Table S4. Cluster comparisons of negative symptom severity in the CHR group only***

| Negative symptom factor/domain | Value Representation Deficit (Clus1) | Generalized Deficit (Clus2) | Hedonic Reactivity Deficit (Clus3) | Test Statistic | Significant Pairwise Differences |
| --- | --- | --- | --- | --- | --- |
| NSI-PR Diminished Motivation & Pleasure Factor | 1.58 (0.85) | 2.17 (0.79) | 1.53 (0.79) | *F*(2,105) = 4.15, *p*  = .018, η_p_^2^ = 0.07 | Clus2 > Clus1 (*p* = .025)  Clus2 > Clus3 (*p* = .031) |
| NSI-PR Diminished Expression Factor | 0.94 (1.00) | 1.61 (1.38) | 0.83 (1.03) | *F*(2,104) = 3.27, *p*  = .042, η_p_^2^ = 0.06 | Clus2 > Clus1 (*p* = .071; *p_uncorrected_* = .024)  Clus2 > Clus3 (*p* = .054; *p_uncorrected_* = .018) |
| Anhedonia | 1.19 (1.11) | 1.75 (1.25) | 1.28 (0.94) | *F*(2,104) = 1.81, *p*  = .170, η_p_^2^ = 0.03 | – |
| Asociality | 1.77 (0.95) | 2.53 (1.03) | 1.62 (1.12) | *F*(2,105) = 4.96, *p*  = .009, η_p_^2^ = 0.09 | Clus2 > Clus1 (*p* = .020)  Clus2 > Clus3 (*p* = .010) |
| Avolition | 1.63 (1.08) | 2.06 (0.86) | 1.62 (0.85) | *F*(2,105) = 1.42, *p*  = .246, η_p_^2^ = 0.03 | – |
| Blunted Affect | 1.08 (1.17) | 1.81 (1.59) | 0.93 (1.17) | *F*(2,104) = 3.10, *p*  = .049, η_p_^2^ = 0.06 | Clus2 > Clus1 (*p* = .092; *p_uncorrected_* = .031)  Clus2 > Clus3 (*p* = .059; *p_uncorrected_* = .020) |
| Alogia | 0.53 (0.80) | 1.06 (1.26) | 0.53 (0.82) | *F*(2,104) = 2.61, *p*  = .079, η_p_^2^ = 0.05 | – |

**Results of the cluster analysis performed without using the HC group as a reference sample**

***Table S5. Clustering index results (using unreferenced scores)***

| Number of clusters | Number of indices proposing this cluster solution |
| --- | --- |
| 2 | 4 |
| **3** | **8** |
| 4 | 6 |
| 5 | 2 |
| 6 | 1 |
| 7 | 0 |
| 8 | 0 |
| 9 | 0 |
| 10 | 2 |

*Note*. One of the 24 examined cluster indices proposed a 1-cluster solution, which fell outside the pre-specified range of 2-10 clusters considered for analysis.

***Cluster plot (using unreferenced scores)***

***Figure S2.*** Cluster plot of 3-cluster solution in Principal Component Space (from the analysis using unreferenced scores). Ellipses represent Euclidean distance from the cluster center.

***Figure S3.* Reward processing profiles of each cluster (from the analysis using unreferenced scores).** Clusters were characterized by a hedonic reactivity deficit (cluster 1), a more generalized deficit across reward processing domains (cluster 2), and a value representation deficit (cluster 3). Diamonds denote mean scores and boxplots indicate the median and interquartile range. The dotted line at *z* = 0 represents the mean value of healthy control reference group to which task scores were *z*-scored.

***Table. S6. Jaccard cluster stability scores from the clusterwise bootstrapping analysis (using unreferenced scores)***

|  | Cluster 1 (*n* = 54) | Cluster 2 (*n* = 35) | Cluster 3 (*n* = 109) |
| --- | --- | --- | --- |
| Jaccard cluster stability score | 0.73 | 0.73 | 0.70 |

***Table S7. Linear discriminant analysis (LDA) classification table (using unreferenced scores)***

| Actual cluster assignment | LDA predicted assignment | | |
| --- | --- | --- | --- |
|  | Cluster 1 | Cluster 2 | Cluster 3 |
| Cluster 1 | 50 | 0 | 4 |
| Cluster 2 | 0 | 31 | 4 |
| Cluster 3 | 0 | 0 | 109 |

*Note.* Linear discriminant analysis overall classification accuracy: 96%

***Table S8. Cluster subgroup characteristics (from the cluster analysis using unreferenced scores)***

|  | Hedonic Reactivity Deficit (Clus1) | Generalized Deficit (Clus2) | Value Representation Deficit (Clus3) | Test Statistic | Significant Pairwise Differences |
| --- | --- | --- | --- | --- | --- |
| ***n (%)*** | 54 (27%) | 35 (18%) | 109 (55%) |  |  |
| ***Demographic variables*** |  |  |  |  |  |
| Clinical Group (CHR/HSC) | 28/26 | 20/15 | 62/47 | χ^2^(2, N = 198) = 0.41, *p* = .813 |  |
| Age | 23.19 (4.36) | 23.60 (4.37) | 24.01 (4.13) | *F*(2,195) = 0.89, *p*  = .422, η_p_^2^ = 0.01 |  |
| Sex (M/F) | 8/46 | 10/25 | 38/71 | χ^2^(2, N = 198) = 7.16, *p* = .028 | Clus3 > Clus1 |
| *Ethnicity/Race* |  |  |  | χ^2^(10, N = 197) = 7.86, *p* = .642 |  |
| Asian | 17% | 23% | 19% |  |  |
| Black | 9% | 20% | 12% |  |  |
| Latinx | 15% | 6% | 11% |  |  |
| Multiracial | 17% | 6% | 8% |  |  |
| Middle Eastern | 4% | 3% | 3% |  |  |
| White-Non-Hispanic | 39% | 43% | 46% |  |  |
| Education (years) | 14.60 (2.46) | 14.03 (2.27) | 14.63 (2.04) | *F*(2,190) = 0.99, *p*  = .374, η_p_^2^ = 0.01 |  |
| ***Clinical variables*** |  |  |  |  |  |
| NSI-PR Diminished Motivation & Pleasure Factor | 1.36 (0.80) | 1.81 (0.76) | 1.48 (0.80) | *F*(2,190) = 3.29, *p_FDR_*  = .059 (*p_uncorrected_* = .039), η_p_^2^ = 0.03 | Clus2 > Clus1 (*p* = .039)  Clus2 > Clus3 (*p* = .111; *p_uncorrected_* = .037) |
| NSI-PR Diminished Expression Factor | 0.67 (0.91) | 1.48 (1.24) | 0.81 (0.90) | *F_W_*(2,72.96) = 5.38, *p_FDR_*  = .025, η_p_^2^ = 0.08 | Clus2 > Clus1 (*p* = .005)  Clus2 > Clus3 (*p* = .016) |
| Anhedonia | 1.14 (1.03) | 1.53 (1.07) | 1.15 (1.02) | *F*(2,189) = 1.86, *p_FDR_*  = .184, η_p_^2^ = 0.02 |  |
| Asociality | 1.50 (1.01) | 2.06 (1.00) | 1.69 (0.96) | *F*(2,190) = 3.22, *p_FDR_*  = .059 (*p_uncorrected_* = .042), η_p_^2^ = 0.03 | Clus2 > Clus1 (*p* = .037) |
| Avolition | 1.37 (0.91) | 1.71 (0.98) | 1.46 (0.99) | *F*(2,190) = 1.34, *p_FDR_*  = .265, η_p_^2^ = 0.01 |  |
| Blunted Affect | 0.77 (1.06) | 1.64 (1.38) | 0.92 (1.05) | *F*(2,188) = 6.79, *p_FDR_*  = .021, η_p_^2^ = 0.07 | Clus2 > Clus1 (*p* = .002)  Clus2 > Clus3 (*p* = .004) |
| Alogia | 0.37 (0.63) | 1.06 (1.22) | 0.47 (0.80) | *F_W_*(2,73.85) = 4.53, *p_FDR_*  = .033, η_p_^2^ = 0.08 | Clus2 > Clus1 (*p* = .012)  Clus2 > Clus3 (*p* = .034) |
| Proxy Primary Negative Symptom Score (*z*-score)^a^ | −0.05 (1.01) | 0.34 (1.00) | −0.09 (0.98) | *F*(2,188) = 2.39, *p*  = .095, η_p_^2^ = 0.03 |  |
| SIPS Positive (Total) | 7.69 (4.63) | 7.62 (4.70) | 7.80 (4.40) | *F*(2,190) = 0.03, *p*  = .974, η_p_^2^ = 0.00 |  |
| CES-D | 17.70 (8.04) | 19.00 (10.20) | 17.35 (9.55) | *F*(2,193) = 0.41, *p*  = .665, η_p_^2^ = 0.00 |  |
| PSS | 28.52 (8.30) | 30.21 (9.82) | 28.93 (8.85) | *F*(2,193) = 0.40, *p*  = .674, η_p_^2^ = 0.00 |  |
| STAI (Trait) | 16.24 (5.17) | 17.41 (6.19) | 16.64 (5.74) | *F*(2,193) = 0.93, *p*  = .912, η_p_^2^ = 0.00 |  |
| *Any DSM-5 Diagnosis (%)* | 72.2% | 77.14% | 72.48% | χ^2^(2, N = 193) = 0.33, *p* = .847 |  |
| Bipolar Disorders | 9.26% | 11.43% | 4.59% | χ^2^(2, N = 198) = 2.43, *p* = .297 |  |
| Depressive Disorders | 29.63% | 28.57% | 32.11% | χ^2^(2, N = 198) = 0.20, *p* = .903 |  |
| Substance Use Disorders | 18.52% | 22.86% | 16.51% | χ^2^(2, N = 198) = 0.72, *p* = .697 |  |
| Anxiety Disorders | 61.11% | 51.43% | 59.63% | χ^2^(2, N = 198) = 0.93, *p* = .628 |  |
| Obsessive-Compulsive Disorders | 7.41% | 11.43% | 9.17% | χ^2^(2, N = 198) = 0.412, *p* = .812 |  |
| Trauma Disorders | 22.22% | 31.43% | 22.94% | χ^2^(2, N = 198) = 1.21, *p* = .547 |  |
| Global Functioning: Social | 7.86 (1.28) | 6.91 (1.38) | 7.61 (1.32) | *F*(2,187) = 5.44, *p*  = .005, η_p_^2^ = 0.06 | Clus2 < Clus1 (*p* = .004)  Clus2 < Clus3 (*p* = .024) |
| Global Functioning: Role | 8.22 (1.24) | 7.55 (1.42) | 7.93 (1.42) | *F*(2,187) = 2.40, *p*  = .094, η_p_^2^ = 0.03 |  |
| NAPLS 1-year Psychosis Conversion Risk Probability | 8.48% (5.54) | 11.65% (7.92) | 7.98% (3.79) | *F*(2,89) = 3.12, *p*  = .049, η_p_^2^ = 0.07 | Clus2 > Clus3 (*p* = .045) |
| NAPLS 2-year Psychosis Conversion Risk Probability | 11.15% (7.14) | 15.16% (10.04) | 10.53% (4.91) | *F*(2,89) = 3.02, *p*  = .054, η_p_^2^ = 0.06 |  |
| Current antipsychotic use | 5.66% | 6.06% | 1.87% | χ^2^(2, N = 193) = 2.13, *p* = .344 |  |
| ***Cognitive Variables*** |  |  |  |  |  |
| Estimated Premorbid Functioning (WRAT-4 Word Reading) | 113.76 (13.90) | 107.73 (15.98) | 110.84 (12.19) | *F*(2,160) = 1.84, *p*  = .164, η_p_^2^ = 0.02 |  |
| *Verbal Learning (HVLT)* |  |  |  |  |  |
| Total score | 2772 (5.13) | 25.27 (5.52) | 27.31 (4.51) | *F*(2,186) = 2.83, *p*  = .062, η_p_^2^ = 0.03 |  |
| Learning rate (Trial 3 – Trial 1) | 2.68 (1.99) | 3.24 (1.73) | 2.61 (1.61) | *F*(2, 186) = 1.68, *p*  = .188, η_p_^2^ = 0.02 |  |
| Processing Speed (BACS Symbol Coding) | 59.91 (11.58) | 58.27 (11.62) | 58.57 (13.53) | *F*(2, 170) = 0.22, *p*  = .806, η_p_^2^ = 0.00 |  |
| ***Task performance*** |  |  |  |  |  |
| Hedonic Reactivity (average IAPS Pleasantness rating) | −0.80 (1.04) | −0.19 (1.00) | 0.46 (0.66) | *Fw*(2,70.30) = 35.79, *p*  < .001, η_p_^2^ = 0.30 | Clus1 < Clus2 (*p* = .019)  Clus1 < Clus3 (*p* < .001)  Clus2 < Clus3 (*p*  = .002) |
| Value Representation (average *k*-delay discounting rate) | 0.99 (0.86) | −0.31 (1.01) | −0.39 (0.68) | *Fw* (2,73.12) = 54.39, *p*  < .001, η_p_^2^ = 0.37 | Clus2 < Clus1 (*p*  < .001)  Clus3 < Clus1 (*p*  < .001) |
| Effort-Cost Computation (EEfRT % Hard task choices^b^) | 0.12 (0.93) | −0.38 (1.09) | 0.06 (0.99) | *F*(2,195) = 3.13, *p*  =.046, η_p_^2^ = 0.03 | Clus2 < Clus1 (*p*  = .065; *p_uncorrected_* = .022)  Clus2 < Clus3 (*p*  = .073; *p_uncorrected_* = .024) |
| Reinforcement Learning (PRLT % Gain condition accuracy^c^) | 0.51 (0.49) | −1.77 (0.83) | 0.32 (0.50) | *Fw*(2,75.25) = 111.90, *p*  < .001, η_p_^2^ = 0.68 | Clus2 < Clus1 (*p*  < .001)  Clus2 < Clus3 (*p*  < .001) |

*Note*. Values reflect Mean (Standard Deviation) unless otherwise indicated. CHR = Clinical High-Risk participants; HSC = Help-Seeking Control participants; NSI-PR = Negative Symptom Inventory – Psychosis Risk; SIPS = Structured Interview for Psychosis-Risk Syndromes; CES-D = Center for Epidemiologic Studies – Depression Scale; PSS = Perceived Stress Scale; STAI = State-Trait Anxiety Inventory; WRAT-4 = Wide-Range Achievement Test – 4; HVLT = Hopkins Verbal Learning Test; BACS = Brief Assessment of Cognition in Schizophrenia; IAPS = International Affective Picture System; EEfRT = Effort Expenditure for Rewards Task; PRLT = Probabilistic Reinforcement Learning Task; *Fw* = Welch’s *F*-statistic; *p_FDR_* = False-Discovery Rate-corrected *p*-value.

^a^Proxy primary negative symptom score is the scaled difference score of N1+N2+N3-G2-G4 SIPS items.

^b^Percentage of hard task choices for trials in the upper half of the reward magnitude range and in the upper (50%-88%) reward probability range.

^c^Percentage of correct choices on the most frequently rewarded gain stimulus pairing across learning trials.

**References**

Benedict, R. H., Schretlen, D., Groninger, L., & Brandt, J. (1998). Hopkins Verbal Learning Test–Revised: Normative data and analysis of inter-form and test-retest reliability. *The Clinical Neuropsychologist*, *12*(1), 43–55.

Bieling, P. J., Antony, M. M., & Swinson, R. P. (1998). The State-Trait Anxiety Inventory, Trait version: Structure and content re-examined. *Behaviour Research and Therapy*, *36*(7–8), 777–788. https://doi.org/10.1016/s0005-7967(98)00023-0

Cannon, T. D., Yu, C., Addington, J., Bearden, C. E., Cadenhead, K. S., Cornblatt, B. A., Heinssen, R., Jeffries, C. D., Mathalon, D. H., McGlashan, T. H., Perkins, D. O., Seidman, L. J., Tsuang, M. T., Walker, E. F., Woods, S. W., & Kattan, M. W. (2016). An Individualized Risk Calculator for Research in Prodromal Psychosis. *American Journal of Psychiatry*, *173*(10), 980–988. https://doi.org/10.1176/appi.ajp.2016.15070890

Charrad, M., Ghazzali, N., Boiteau, V., & Niknafs, A. (2014). NbClust: an R package for determining the relevant number of clusters in a data set. *Journal of statistical software*, *61*, 1-36.

Cohen, S., Kamarck, T., & Mermelstein, R. (1983). A global measure of perceived stress. *Journal of Health and Social Behavior*, *24*, 385–396. https://doi.org/10.2307/2136404

Cornblatt, B. A., Auther, A. M., Niendam, T., Smith, C. W., Zinberg, J., Bearden, C. E., & Cannon, T. D. (2007). Preliminary findings for two new measures of social and role functioning in the prodromal phase of schizophrenia. *Schizophrenia Bulletin*, *33*(3), 688–702. https://doi.org/10.1093/schbul/sbm029

Culbreth, A. J., Moran, E. K., & Barch, D. M. (2018). Effort-cost decision-making in psychosis and depression: Could a similar behavioral deficit arise from disparate psychological and neural mechanisms? *Psychological Medicine*, *48*(6), 889–904. https://doi.org/10.1017/S0033291717002525

First, M. B., Williams, J. B., Karg, R. S., & Spitzer, R. L. (2015). Structured clinical interview for DSM-5—Research version (SCID-5 for DSM-5, research version; SCID-5-RV). *Arlington, VA: American Psychiatric Association*, *1–94*.

Gold, J. M., Waltz, J. A., Matveeva, T. M., Kasanova, Z., Strauss, G. P., Herbener, E. S., Collins, A. G. E., & Frank, M. J. (2012). Negative symptoms and the failure to represent the expected reward value of actions: Behavioral and computational modeling evidence. *Archives of General Psychiatry*, *69*(2), 129–138. https://doi.org/10.1001/archgenpsychiatry.2011.1269

Gray, J. C., Amlung, M. T., Palmer, A. A., & MacKillop, J. (2016). Syntax for calculation of discounting indices from the monetary choice questionnaire and probability discounting questionnaire. *Journal of the Experimental Analysis of Behavior*, *106*(2), 156–163.

Hennig, C. (2007). Cluster-wise assessment of cluster stability. *Computational Statistics & Data Analysis*, *52*(1), 258–271. https://doi.org/10.1016/j.csda.2006.11.025

Hennig, C. (2008). Dissolution point and isolation robustness: Robustness criteria for general cluster analysis methods. *Journal of Multivariate Analysis*, *99*(6), 1154–1176. https://doi.org/10.1016/j.jmva.2007.07.002

Hennig, C. (2020). *fpc: Flexible procedures for clustering (Version 2.2-9)*.

Janssen, I., Krabbendam, L., Bak, M., Hanssen, M., Vollebergh, W., de Graaf, R., & van Os, J. (2004). Childhood abuse as a risk factor for psychotic experiences. *Acta Psychiatrica Scandinavica*, *109*(1), 38–45. https://doi.org/10.1046/j.0001-690X.2003.00217.x

Keefe, R. S. E., Goldberg, T. E., Harvey, P. D., Gold, J. M., Poe, M. P., & Coughenour, L. (2004). The Brief Assessment of Cognition in Schizophrenia: Reliability, sensitivity, and comparison with a standard neurocognitive battery. *Schizophrenia Research*, *68*(2), 283–297. https://doi.org/10.1016/j.schres.2003.09.011

Kirby, K. N., Petry, N. M., & Bickel, W. K. (1999). Heroin addicts have higher discount rates for delayed rewards than non-drug-using controls. *Journal of Experimental Psychology: General*, *128*(1), 78.

Lang, P. J., Bradley, M. M., & Cuthbert, B. N. (1997). International affective picture system (IAPS): Technical manual and affective ratings. *NIMH Center for the Study of Emotion and Attention*, *1*(39–58), 3.

Maxwell, M. E. (1992). Family Interview for Genetic Studies (FIGS): A manual for FIGS. *Bethesda, MD: Clinical Neurogenetics Branch, Intramural Research Program, National Institute of Mental Health*.

R Core Team. (2020). *R: A Language and Environment for Statistical Computing*. R Foundation for Statistical Computing. https://www.R-project.org/

Radloff, L. S. (1977). The CES-D Scale: A self-report depression scale for research in the general population. *Applied Psychological Measurement*, *1*, 385–401. https://doi.org/10.1177/014662167700100306

Strauss, G. P., Walker, E. F., Pelletier-Baldelli, A., Carter, N. T., Ellman, L. M., Schiffman, J., ... & Mittal, V. A. (2023). Development and Validation of the Negative Symptom Inventory-Psychosis Risk. *Schizophrenia Bulletin*, sbad038.

Treadway, M. T., Buckholtz, J. W., Schwartzman, A. N., Lambert, W. E., & Zald, D. H. (2009). Worth the ‘EEfRT’? The Effort Expenditure for Rewards Task as an Objective Measure of Motivation and Anhedonia. *PLOS ONE*, *4*(8), e6598. https://doi.org/10.1371/journal.pone.0006598

Wilkinson, G. S., & Robertson, G. J. (1993). Wide range achievement test 4. *Journal of Clinical and Experimental Neuropsychology*.
